# Recent Advances in the Use of Polyhydroyalkanoates in Biomedicine

**DOI:** 10.3390/bioengineering6030082

**Published:** 2019-09-12

**Authors:** Alejandra Rodriguez-Contreras

**Affiliations:** Department of Materials Science and Metallurgical Engineering, Universitat Politècnica de Catalunya (UPC), Escola d’Enginyeria de Barcelona Est (EEBE), Eduard Maristany 10-14, 08930 Barcelona, Spain; sandra8855@hotmail.com; Tel.: +34-651-569-562

**Keywords:** polyhydroxyalkanoates, biomedicine, biomaterials, Poly(3-hydroxybutyrate), tissue engineering, wound healing, delivery system, poly(3-hydroxybutyrate-co-3-hydroxyvalerate) (PHVB), poly(3-hydroxybutyrate-co-4-hydroxybutyrate)

## Abstract

Polyhydroxyalkanoates (PHAs), a family of natural biopolyesters, are widely used in many applications, especially in biomedicine. Since they are produced by a variety of microorganisms, they possess special properties that synthetic polyesters do not have. Their biocompatibility, biodegradability, and non-toxicity are the crucial properties that make these biologically produced thermoplastics and elastomers suitable for their applications as biomaterials. Bacterial or archaeal fermentation by the combination of different carbohydrates or by the addition of specific inductors allows the bioproduction of a great variety of members from the PHAs family with diverse material properties. Poly(3-hydroxybutyrate) (PHB) and its copolymers, such as poly(3-hydroxybutyrate-co-3-hydroxyvalerate) (PHVB) or poly(3-hydroxybutyrate-co-4-hydroxybutyrate) (PHB4HB), are the most frequently used PHAs in the field of biomedicine. PHAs have been used in implantology as sutures and valves, in tissue engineering as bone graft substitutes, cartilage, stents for nerve repair, and cardiovascular patches. Due to their good biodegradability in the body and their breakdown products being unhazardous, they have also been remarkably applied as drug carriers for delivery systems. As lately there has been considerable and growing interest in the use of PHAs as biomaterials and their application in the field of medicine, this review provides an insight into the most recent scientific studies and advances in PHAs exploitation in biomedicine.

## 1. Introduction

Synthetic plastics are used in many different applications, as they are a family of versatile materials. However, there is a global awareness of the environmental impact of these fossil-based polymers. At the same time, there is growing recognition that organic matter of biological origin can be a worthy alternative [1]. In this regard, natural polymers or biopolymers show many advantages relative to petrochemical materials, as they are biodegradable and produced from renewable sources. Furthermore, due to their similarity to the native natural environment, their biopolymer functions show good biological performance and adaptability, and adequate body reaction [2]. This makes them very attractive for their application not only in biomedicine but also in other fields such as pharmacology and biotechnology [1]. In biomedicine as the theoretical branch of medicine that applies the principles of biology, biochemistry, and biophysics to medical research and practice, the combination of synthetic and natural polymers is frequently used [3,4,5,6].

**Figure 1:**
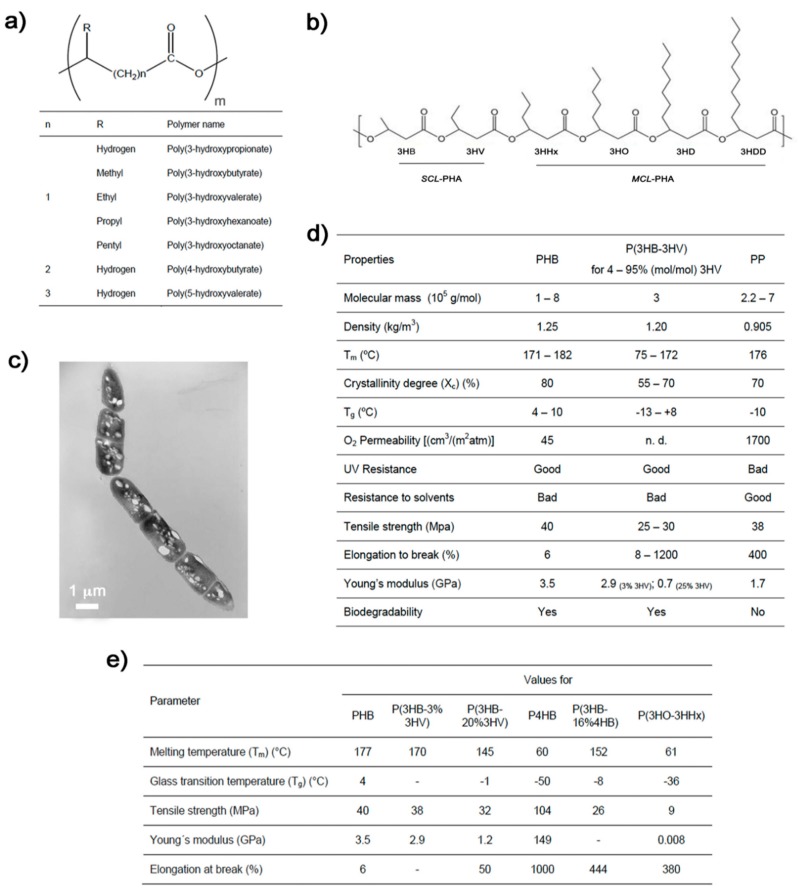
(**a**) Chemical structure of the polyhydroxyalkanoates (PHA) biopolymer family, the monomer number m range from 100 to 30,000 [12]. (**b**) Some commonly synthesized scl-PHA monomers (scl-HA) and mcl-PHA monomers (mcl-HA). 3HB: 3-hydroxybutyrate, 3HV: 3-hydroxyvalerate, 3HHx: 3-hydroxyhexanoate, 3HO: 3-hydroxyoctanoate, 3HD: 3-hydroxydecanoate, 3HDD: 3-hydroxydodecanoate. (**c**) Transmission electron microscopy micrograph of *Bacillus megaterium* uyuni S29 after 4 h of fermentation showing PHB granules as refractile inclusion bodies [28]. (**d**) Some physical, thermal, chemical, and mechanical properties of PHB and poly(3-hydroxybutyrate-co-3-hydroxyvalerate) (PHBV) compared to those of the petrol-based polypropylene (PP) [12,29]. (**e**) Table of properties of some PHAs members and copolymers [12,29].

Polyhydroxyalkanoates (PHAs) are a big family of naturally produced polyesters. Chemically, they are linear polymers composed of hydroxyalkanoate units as their basic structure (Figure 1a). These biopolymers are accumulated within the cytoplasm of diverse microorganisms under conditions of nutrient depletion and in the presence of an excess of carbon source [7,8,9]. They appear as granules and function as carbohydrate and energy storage (Figure 1c). PHAs can be produced by biotechnological processes via bacterial and archaeal fermentation. The members of the PHA family differ widely in their structure and properties (Figure 1a,d,e) depending on the producing microorganism, biosynthesis conditions, and type of carbon source used in the production process [8,9,10]. In general, PHAs are thermoplastic or elastomeric, and their sufficiently high molecular mass provides them with properties similar to those of conventional petrochemical polymers (Figure 1d) [11,12,13,14]. More specifically, they can be classified depending on their monomeric composition: short-chain-length PHA (*scl*-PHA), consisting of 3 to 5 carbon atoms per monomer; medium-chain-length PHA (*mcl*-PHA), with 6 to 14 carbon atoms; and the rather rare group of long-chain-length PHA (*lcl*-PHA), which presents more than 14 carbon atoms [15,16]. The vast majority of microorganisms synthesize either *scl*-PHAs containing primarily 3-hydroxybutyrate (3HB) units or *mcl*-PHAs containing 3-hydroxyhexanoate (3HHx), 3-hydroxyoctanoate (3HO), 3-hydroxydecanoate (3HD), and 3-hydroxydodecanoate (3HHD) as the major monomers [7,15]. While *scl*-PHAs are crystalline and feature typical thermoplastic properties, *mcl*-PHA resins resemble elastomers and latex-like materials with typically low glass transition temperature and lower molecular mass if compared to *scl*-PHA [15,17,18].

Poly(3-hydroxybutyrate) (PHB) is the most frequently occurring PHA member and is a linear, unbranched homopolymer consisting of (R)-3-hydroxybutyric acid units. When extracted from bacterial biomass, PHB tends to crystallize [19]. Although its applications are limited mainly by its high crystallinity and brittleness, which reduce its flexibility and ductility, PHB can be modified by simply physical blending or chemical alteration to fine-tune its mechanical properties [10,20]. Another strategy to modify its mechanical properties is by copolymerization via bacterial fermentation using different precursors (Figure 1d). For instance, a common PHB copolymer, poly(3-hydroxybutyrate-co-3-hydroxyvalerate) (PHBV), is characterized as less crystalline and more flexible than PHB itself [21], and its properties can be varied according to the 3-hydroxyvalerate (3HV) content in the structure (Figure 1d) [22]. PHBV is usually produced by adding valeric acid to the fermentation medium [23]. Poly(3-hydroxybutyrate-co-4-hydroxybutyrate) (PHB4HB) copolymer is another of the most well-known members of the PHA family. With higher 4-hydroxybutyric acid (4HB) content, PHA is more elastomeric and with outstanding elongation at break (Figure 1d) [24]. These PHAs members together with poly(3-hydroxybutyrate-co-3-hydroxyvalerate-co-3-hydroxyhexanoate) (PHBVHHx) represent the most commonly applied PHAs in biomedicine [25].

Current research on PHAs focuses on subjects such as gaining a better understanding of the mechanisms related to their biosynthesis, or how to modulate PHAs properties for different applications. The development of natural and recombinant microorganisms to efficiently produce PHAs and the finding of alternative raw materials that lead their production to more competitive costs are also important research topics [26,27].

PHAs show major advantages compared with traditional synthetic polymers. However, it is because of their biodegradability, biocompatibility, and non-toxicity that they are especially appealing materials for biomedical applications. Furthermore, an additional benefit is their unchanged local pH value during degradation. This makes them well tolerated by cells and the immune system compared to other polymers clinically used such as poly(lactide-co-glycolide) (PLGA), poly(ε-caprolactone) (PCL), poly(glycolic acid) (PGA), and poly(lactic acid) (PLA) [20]. In the last decades, there has been an increase in PHAs exploitation in biomedicine. Therefore, this review is an attempt to summarize the most important advances published in the last few years on the use of microbially originated PHAs used in this field.

## 2. Tissue Engineering

Tissue engineering is an interdisciplinary field of research focused on the creation of vital tissues by a combination of biomaterials, cells, and bioactive molecules, aiming to repair damaged or diseased tissues and organs [30]. Tissues can be classified as hard tissue substitutes, such as bone and cartilage, or soft tissues, such as vascular and skin grafts [31]. The biomaterial used must have two crucial features to function as tissue repairer: to possess mechanical properties for supporting the organ during new tissue regeneration, and enhanced surface topography to allow efficient cell adhesion and proliferation. In this regard, engineered scaffolds are designed to closely mimic the topography, spatial distribution, and chemical environment corresponding to the native extracellular matrix of the intended tissue in order to support cell growth and differentiation [32]. PHAs constitute a great alternative for tissue engineering due to their versatility regarding their mechanical properties, combined with great biocompatibility with minimal tissue toxicity and degradability. Thus, PHAs have been exploited for the replacement and healing of both hard and soft tissues in tissue engineering to repair cartilage, cardiovascular tissues, skin, bone marrow, and nerve conduits [22,33,34,35].

### 2.1. Hard Tissue

#### 2.1.1. Bone Tissue Engineering

Bone tissue engineering refers to the regeneration of new bone by providing mechanical support while inducing cell growth. For this application, hydroxyapatite (HA), inorganic substances, hydrogels, and even other biocompatible polymers are used to blend with PHAs to optimize their compressive elastic modulus and maximum stress. For instance, Degli Esposti et al. [36] very recently published the exploitation of a mixture of PHB with HA particles for the development of bio-resorbable porous scaffolds for bone tissue regeneration. The osteoinductivity and osteoconductivity of the bioactive scaffolds were attained mainly due to the incorporation of HA. By combining CaCO_3_-mineralized piezoelectric with PHB- and PHBV-based scaffolds, Chernozem et al. [37] elaborated PHA biocomposites that provided biodegradability and stimulated bone tissue repair. The presence of mineral led to a pronounced apatite-forming behavior of the biodegradable PHAs scaffolds, and this turned out to stimulate the growth of the bone tissue. A more complex system is the one produced by Meischel et al. [38], who evaluated the response of bone to PHA composite implants in the femora of growing rats. Composites were constituted by PHB with zirconium dioxide, Herafill^®^ (calcium sulfate, calcium carbonate, triglycerides, and gentamicin; produced by Hereus), and Mg-alloy WZ21. Longitudinal observation of the bone reaction at the implant site and resorption of the implanted pins were monitored, and the results showed that PHB composited with zirconium dioxide and 30% Herafill possessed the highest values of bone accumulation. The authors concluded that the mechanical properties (elastic modulus, tensile strength, and strain properties) of PHB composites in these conditions were close to that of bone.

**Figure 2:**
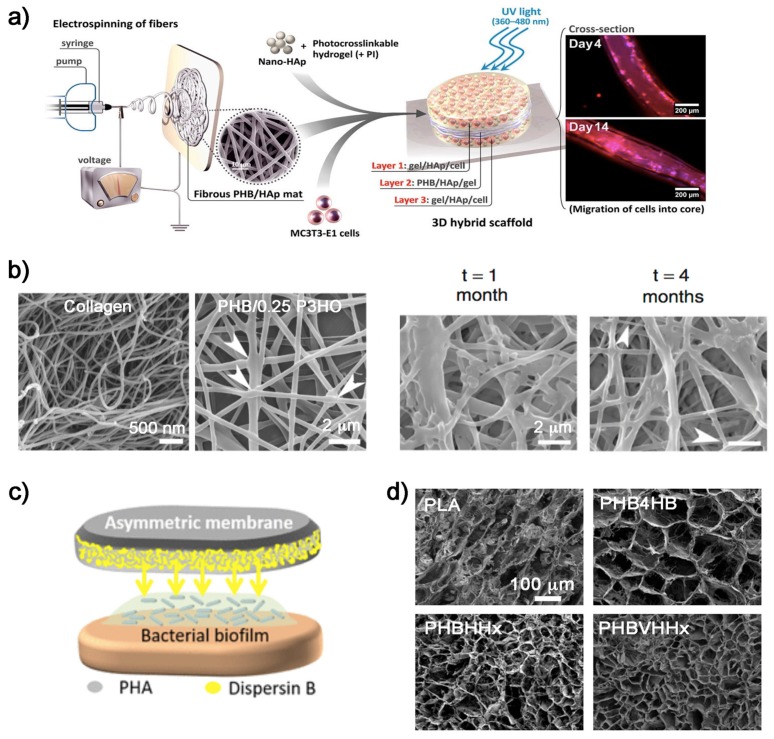
PHAs for tissue engineering: (**a**) Scheme of the published study of Sadat-Shojai et al. [39] where a cell-laden tri-layered scaffold of PHB with hydroxyapatite (HA) was performed to enhance bone regeneration in vivo. (**b**) Scanning electron microscopy (SEM) micrographs of PHB/P3HO scaffolds where the electrospun fibers with a ratio blend of 1:0.25 provided structures more similar to collagen natural fibers. Biopolymeric fibers after hydrolytic degradation [41]. (**c**) Scheme of the asymmetric PHA membranes entrapping an anti-biofilm protein (dispersin B) for wound healing [49]. (**d**) SEM micrographs of biopolymers scaffolds from Li et al. [50]. The biopolymer structures displayed different pore sizes where stem cells were loaded into, and the PHBVHHx ones exhibited the highest cell attachment.

Hydrogels can be used to create scaffolds with a well-interconnected porous structure. However, they provide poor mechanical stability and very low bioactivity, failing to create suitable constructs for bone tissue engineering. In order to improve the mechanical stability of hydrogels, Sadat et al. [39] developed a scaffold system based on combining a mix of biodegradable PHB and HA with a protein-based hydrogel in a single tri-layered scaffold. These scaffolds provided high strength, had the ability to encapsulate cells, and enhanced bone cell adaptability (Figure 2a).

In their study, Ding et al. [40] mixed the natural polyester PHB with the synthetic polyester PCL. They fabricated PHB/PCL/58S sol-gel bioactive glass hybrid scaffolds by electrospinning of the polymers and inorganic substances. The combination of the high stiffness of PHB, the flexibility of PCL, and the bioactivity of 58S bioactive glass in one single fibrous structure showed potential for using in bone tissue engineering integration. The composite enhanced the primary biological response of osteoblast-like cells and their viability, and significantly increased alkaline phosphatase enzyme activity.

#### 2.1.2. Cartilage

Tissue engineering of cartilage provides promising strategies for the regeneration of damaged articular cartilage. There are significant challenges, since current surgical procedures are unable to restore normal cartilage function. It is important to create an alternative that matches the long-term mechanical stability and durability of this native hard tissue [41]. Some recent studies demonstrated that the use of PHAs can be a solution. Ching et al. [41] produced diverse blends of PHB with poly(3-hydroxyoctanoate) (P3HO) as biodegradable polymer scaffolds. By studding different ratios of both polymers, they optimized their structure, stiffness, degradation rates, and biocompatibility. At a polymer rate (PHB/P3HO) of 1:0.25, the blend closely mimicked the collagen fibrillar meshwork of native cartilage and attained the stiffness of native articular cartilage (Figure 2b). They concluded that by fine tuning the ultrastructure and mechanical properties using different blends, these two polymers allowed the production of a cartilage repair kit for clinical use and the reduction of the risk of developing secondary osteoarthritis. More recently, Toloue et al. [42] evaluated the mechanical properties and cell viability of a mix of PHB with 3% chitosan reinforced with alumina as a scaffold for cartilage reparation. The presence of alumina nanowires significantly increased the tensile strength of PHB and PHB/chitosan scaffolds. In vitro studies showed that chondrocyte cells spread more on the composite than on pure PHB scaffolds. The authors concluded that the electrospun scaffold of PHB with chitosan and 3% alumina had the potential to be applied in cartilage tissue engineering.

### 2.2. Soft Tissue

#### 2.2.1. Cardiac Tissue Engineering

Cardiac tissue engineering is currently a prime focus of research because of an enormous clinical need. *Mcl*-PHAs have demonstrated exceptional properties for cardiac tissue engineering applications. They are more elastic than other members of their family, showing an elastomeric nature, higher glass transition temperatures, and the potential to integrate with the myocardial network and be conjugated with bioactive molecules, such as vascular endothelial growth factor, to further increase cellular attachment, viability, and proliferation [18].

Guo et al. [35] summarized the recent use of P4HB as a promising biomaterial for applications in cardiac tissue engineering such as congenital heart defects, heart valves, and vascular grafts. The versatile material is also used in other applications as an absorbable monofilament for sutures, and hernia, tendon, and ligament repair, among others. Bagdadi et al. [43] used P3HO as a potential material for cardiac tissue engineering. They fabricated P3HO-based multifunctional cardiac patches with mechanical properties that were close to those of cardiac muscle. Furthermore, they were shown to be as good as collagen in terms of cell viability, proliferation, and adhesion. Likewise, Constantinides et al. [18] used *mcl*-PHAs for this application. They first produced the *mcl*-PHAs by bacterial fermentation with *Pseudomonas mendocina* CH50 using glucose as the sole carbon source under nitrogen limiting conditions. Then, the obtained *mcl*-PHAs were reinforced with PCL (5%) to produce thin films. The blended structures were implanted in post mortem murine heart in situ. The composites demonstrated possessing a great potential for maximizing tissue regeneration in myocardial infarction. Besides this study, there was research using PHBVHHx [25] in the form of membranes and PHB4HB [44] for the production of cardiac patches. These studies were carried out with stem cells of different origin.

Valvular heart diseases are the third leading cause of cardiovascular disease. Thus, heart valve tissue engineering (HVTE) has appeared as an important strategy to treat these disorders. Ideally, a designed construct should withstand the native dynamic mechanical environment, guide the regeneration of the diseased tissue, and more importantly, have the ability to grow with the patient’s heart [45]. Xue et al. [45] summarized different types of synthetic biodegradable elastomers that have been explored for HVTE. Referring to a published work of Chen et al. [46], they specify that this class of elastomers, the PHAs, are generally stronger than polyurethane-based elastomers and more suitable to work under dynamic conditions such as those of cardiovascular tissue.

#### 2.2.2. Wound Healing

The need for novel materials in the effective regeneration of injured skin is a serious concern in reconstructive medicine [47]. Many natural (collagen, alginic acid, hyaluronic acid, chitosan, fucoidan) and synthetic (teflon, polyurethanes, methyl methacrylate) polymers are being used in the preparation of artificial dressing materials for wound healing applications [48]. This complex application requires that the biomaterial fulfills the functions of healthy skin, which has an antimicrobial effect, promotes moist wound environment, permits gaseous exchange, provides mechanical protection, and is sufficiently elastic to fit the wound shape [47]. The PHA family of biopolymers has also extended in this novel medical area. One major factor inhibiting natural wound-healing processes is bacterial infection, especially in chronic wounds [49]. There are studies on wound healing with antibiotic delivery systems and applying PHAs as a remedy. For instance, Marcano et al. [49] optimized the micro/nano-structure of a wound dressing in order to obtain a more efficient antibiofilm protein-release profile for biofilm inhibition and/or detachment. Thus, they developed a three-dimensional (3D) substrate based on asymmetric PHA membranes to entrap an antibiofilm protein (Figure 2c). Similarly, the team of Volova [47] constructed wound dressings from PHB4HB membranes for skin wound repair and evaluated their effectiveness in experiments with laboratory animals. The nonwoven membranes of PHB4HB carried the culture of allogenic fibroblasts. The use of the biopolymer reduced inflammation, enhanced the angiogenic properties of the skin, and facilitated the wound healing process.

#### 2.2.3. PHAs for Organ Tissues

PHBVHHx is considered a promising PHA member for the growth of stem cells, and certain studies utilized it as biomaterial for the preparation of three-dimensional supportive scaffolds for organ tissue. In some works [50,51], PHBVHHx films and scaffolds were developed and loaded with mesenchymal stem cells from human umbilical cord (UC-MSCs) to recover injured liver. Biopolymer scaffolds were transplanted into liver-injured mice, and the results demonstrated that the PHA scaffold significantly promoted the recovery of injured liver and could be used for liver tissue engineering. In the case of the work by Li et al. [50], differences between PHBVHHx and some other commonly used biopolymers such as PLA, PHB4HB, and PHBHHx were examined by loading them with stem cells into their scaffolds (Figure 2d). They concluded that the PHBVHHx structures exhibited the highest cell attachment and, when loaded with mesenchymal stem cells, significantly improved the recovery of injured liver.

PHAs have also been used in tendon healing. In order to improve the initial biomechanical repair strength of tendon tears at risk of failure, Tashjian et al. [52] produced a bioresorbable scaffold to reinforce the suture-tendon interface in rotator cuff repairs. A study of cyclic and ultimate failure properties of PHA mesh was conducted, obtaining better mechanical results than in the control condition (without the reinforcement).

As a hard tissue, Findrik et al. [31] exploited a blend of PLA and PHB to use it as a tubular substitute for urethra replacement. They dealt with the combination of both polymers to provide stabile conditions during the engineering of the replacement by adjusting material degradation and viscosity. By using a 3D printing process, a cubic sample representing basic scaffold structures and a tubular one serving as urethra substitution were designed.

## 3. Drug Delivery Systems

One of the key reasons for the common use of the PHA biopolymer family as drug carriers is their biodegradability under different environments. A vast number of microorganisms secrete extracellular PHA-hydrolyzing enzymes (PHA depolymerases and other enzymes) to degrade PHA polymers into oligomers and monomers, which subsequently act as nutrients inside the cells [53,54,55]. PHAs typically degrade by hydrolytic and bacterial depolymerase mechanisms over 52-plus weeks in vivo [56]. Furthermore, there are studies that compare PHAs biodegradability with that of other synthetic or semisynthetic polymers. Gil-Castell et al. [57] compared the durability of PLGA, polydioxanone (PDO), polycaprolactone (PCL), and PHB scaffolds. Results showed that for long-term applications, PCL and PHB were more appropriate materials than PLGA and PDO, which could be used in short-term applications. Regarding their biodegradability in ultra-pure water and phosphate buffer solution at 37 °C, the PHB molar mass progressively decreased, reaching almost 50% after 650 days of immersion. However, PHAs’ biodegradability depends on different factors such as the composition of the biopolymer, its stereo regularity, crystallinity (degradability decreases as the overall crystallinity increases), molecular mass (biopolymers are generally biodegraded more rapidly when their molecular mas is lower), and environmental conditions (temperature, moisture level, pH, and nutrient supply) [58]. This makes this biopolymer family especially appealing for delivery systems, since the controllable retarding properties of systems based on PHAs can be modulated mainly by their molecular mass and copolymer composition. Moreover, PHAs have already demonstrated a significant impact on the drug bioavailability, better encapsulation, and less toxicity of biodegradable polymers [59].

In the literature, several reviews of the use of PHAs as carriers in biomedine can be found. They embrace the shape of particles, spheres, micelles, liposomes, vesicles, or capsules as therapeutic delivery carriers. For instance, Masood et al. [60] reviewed the current implications of encapsulation of anticancer agents within PHAs, PLGA, and cyclodextrin-based nanoparticles to precisely target the tumor site. The recent scientific developments in the preparation of functionalized PHAs, PHA-drug and PHA-protein conjugates, multifunctional PHA nanoparticles, and micelles as well as biosynthetic PHA particles for drug delivery were reviewed by Michalak et al. [61]. The recent advances of using PHA-based nano-vehicles as therapeutic delivery carriers were summarized by Li and Loh [59]. Pramual et al. [62] developed and investigated nanoparticles of PHAs as carriers of a hydrophobic photosensitizer for photodynamic therapy. Besides these reviews, a patent has been published on the fabrication of a delivery system comprising *scl*-PHA nanoparticles having an anticancer drug encapsulated for oral administration [63]. Also, a similar study on the production of PHB and PHB/poly(ethylene glycol) (PEG)-based microparticles loaded with antitumor drugs by the spray-drying technique was recently published [64]. Apart from these, there are not many more new studies in which PHAs spherical shape structures are considered for drug delivery.

**Figure 3:**
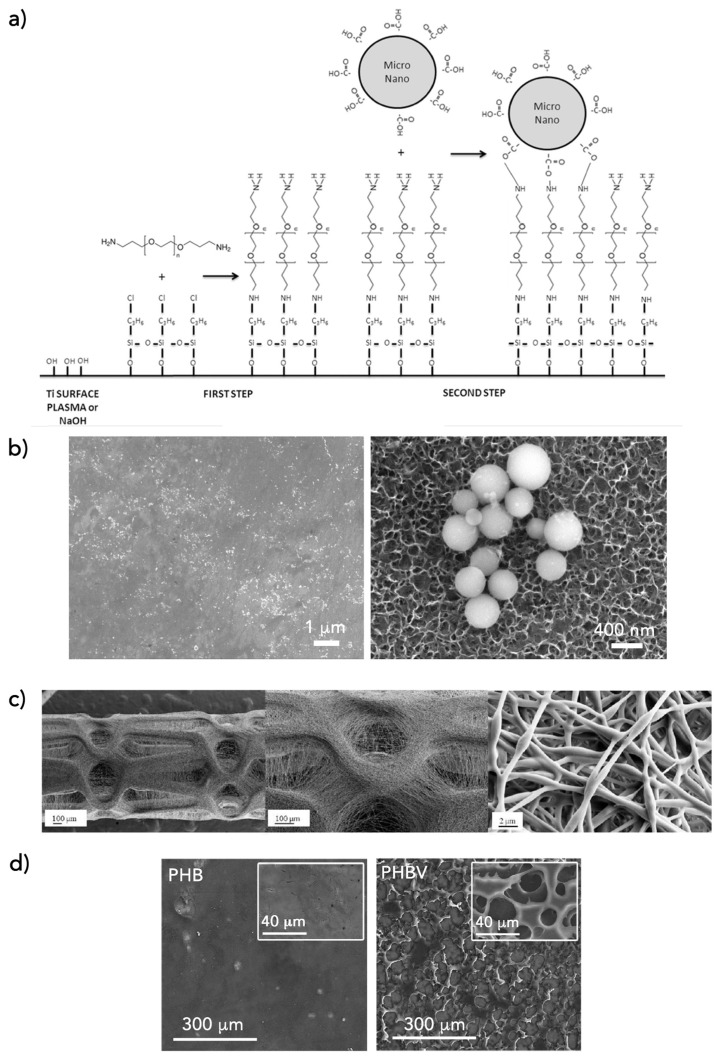
PHAs as drug delivery systems. (**a**) Scheme of the chemical reaction for adhesion of PHB micro- and nano-spheres on Ti surfaces: activation of the Ti surfaces (by plasma or NaOH treatment), silanization with the alkoxysilane 3-chloropropyltriethoxysilane (CPTES), covalent bounding with difunctionalized poly(ethylene glycol) (PEG), and covalent bonding with doxycycline-loaded PHB-spheres. (**b**) Field emission scanning electron microscopy micrographs of Ti surfaces with doxycycline-loaded spheres of PHB [66]. (**c**) SEM images of direct coatings of paclitaxel loaded P(3HB-co-95 mol% 4HB) nanofibers onto a metal stent (40×, 100×, and 5000×) [67]. (**d**) FESEM images at different magnifications of PHB and PHBV matrixes that totally coated Ti surfaces [69].

Manero’s group has been working in the exploitation of PHAs in biomedicine, and they have recently published some studies focused on the application of PHAs as therapeutic delivery carriers. They produced antibiotic (doxycycline)-loaded micro- and nano-particles of PHB with different methodologies [65]. The produced carriers were capable of diffusing the active principle from the material to the media, creating a bacteria-free protective region. Later, new strategies for combining the antibacterial properties of doxycycline-loaded PHB micro- and nano-spheres on titanium (Ti) were developed to obtain implant surfaces with antibacterial activity [66]. Furthermore, they studied a novel approach to benefit the synergistic effects of antifouling PEG together with doxycycline-loaded PHB spheres (Figure 3a,b).

The use of PHAs for delivery systems has been studied with different structures. For instance, Lee et al. [67] developed a system of drug-containing PHA fibers that can be electrospun directly onto a metal stent in order to form a biocompatible coating (Figure 3c). PHAs have been used as matrixes to construct release formulations of antibiotic delivery, providing them with antimicrobial, antifungal, anti-biofilm, anti-inflammatory and virucidal properties dependent on the conjugated/enclosed therapeutic agent. Manero’s group has exploited PHAs matrixes as coatings with an antibacterial delivery effect [68,69,70]. Aiming to obtain antimicrobial surfaces to prevent implant infections, they studied different strategies for developing antibacterial coatings on Ti and Tantalum (Ta). The surface of the biometals was coated with different PHAs (PHB, PHBV, and PHB4HB) using a dip-coating technique. Water-in-oil PHAs emulsions with the bioactive agents were produced to use them as coating fluids. The systems designed for drug delivery not only proved to assure the elimination of the first stage of bacterial biofilm formation (bacterial adhesion), but also their proliferation, since the biopolymer coating with antibiotic was able to degrade with time under physiological conditions, thus guaranteeing a controlled drug release over time (Figure 3d).

Complex systems for drug delivery, such as the one published by Timin et al. [71], have also been developed. In this example, the authors deposited polymer and hybrid microcapsules, which were used as drug carriers, onto polymer microfiber scaffolds of PCL, PHB, and PHB doped with the conductive polyaniline (PANi). The immobilization of the microcapsules (loaded with bioactive molecules) onto the scaffold surfaces enabled multimodal triggering by physical and biological stimuli, providing the controllable release of the drug from the scaffolds. PHB and PHB-PANi scaffolds promoted the adhesion of mesenchymal stem cells compared to that of the PCL scaffolds. With this methodology, they provided a way to incorporate bioactive compounds onto polymer scaffolds, which makes these multimodal materials suitable for personalized drug therapy and bone tissue engineering.

## 4. Conclusions

In the field of biomedicine, biopolymers show many advantages that make them superior to synthetic polymers, predominately because of their natural origin. PHAs represent a big family of biologically produced polymers that show common properties such as biocompatibility, biodegradability, and non-toxicity. These properties together with the ease of PHAs for tuning and adapting their mechanical properties, either by combination with other substances or by copolymerization in the biotechnological production process, make them very attractive for their application in different sectors, especially biomedicine. In the last few years, PHAs have been studied to be used in tissue engineering for hard and soft tissue replacement, and as therapeutic delivery carriers. According to the studies presented in this review and the successful results discussed, given the versatile properties that can be provided by PHAs and the need to continue improving biomedical solutions, PHAs will most likely continue to be investigated as an appealing alternative, penetrating the biomedical market in a not-too-distant future.
